# The Challenging Differentiation of Psoriatic Arthritis from Other Arthropathies and Nonspecific Arthralgias in Patients with Psoriasis: Results of a Cross-Sectional Rheumatologic Assessment of a Large Dermatologic Cohort

**DOI:** 10.3390/jcm12186090

**Published:** 2023-09-21

**Authors:** Alberto Floris, Cristina Mugheddu, Leonardo Sichi, Martina Dessì, Jasmine Anedda, Alessia Frau, Andrea Pau, Simone Aldo Lari, Jessica Sorgia, Laura Li Volsi, Maria Teresa Paladino, Mattia Congia, Elisabetta Chessa, Maria Maddalena Angioni, Caterina Ferreli, Matteo Piga, Laura Atzori, Alberto Cauli

**Affiliations:** 1 Department of Medical Sciences and Public Health, University of Cagliari, 09042 Monserrato, Italy; 2 Rheumatology Unit, Azienda Ospedaliero Universitaria di Cagliari, 09042 Monserrato, Italy; 3 Dermatology Unit, Azienda Ospedaliero Universitaria di Cagliari, 09123 Cagliari, Italy

**Keywords:** psoriasis, psoriatic arthritis, diagnosis, differential diagnosis, multidisciplinary approach

## Abstract

Aiming to identify the potential challenges in the classification of musculoskeletal manifestations in patients with psoriasis (PsO), this study analyzed the outcomes of a cross-sectional rheumatologic assessment of 1057 PsO patients. In total, 209 had a previous diagnosis of psoriatic arthritis (PsA). Out of the remaining 848 subjects, 293 (35%) were classified as suspected PsA cases according to the rheumatologist’s judgment and/or Early PsA Screening Questionnaire score (EARP) ≥ 3. However, only 14% received a PsA diagnosis, 49% had a PsA-alternative diagnosis, and the remaining 37% had nonspecific arthralgias. Most of the newly diagnosed PsA patients had a symptoms duration ≥1 year (72%) and moderate disease activity (55%) with active oligoarthritis (85%), dactylitis, or enthesitis (35%) as the most frequent clinical pattern. The most frequent PsA-alternative diagnoses were osteoarthritis and fibromyalgia (44% and 41%). The only factors with significant (*p* < 0.05) utility in discriminating PsA from other diseases and nonspecific arthralgias were young age and EARP score with a history of morning stiffness, swollen joints, or dactylitis. These results demonstrated a high prevalence of suspected musculoskeletal symptoms in PsO patients, with, however, only a small proportion due to PsA. Close collaboration between the dermatologist and rheumatologist plays a crucial role in the differential diagnosis of PsA, as well as in monitoring nonspecific arthralgias for the potential transition to overt PsA.

## 1. Introduction

Psoriatic arthritis (PsA) is a chronic inflammatory disease characterized by wide clinical heterogeneity due to its variable association with different domains, including skin and nail psoriasis (PsO), peripheral arthritis, enthesitis, dactylitis, and spondylitis. PsA affects up to 30% of patients with PsO, with the joint disease following the skin manifestations in about 80% of cases [1].

If not diagnosed early and adequately treated, PsA may result in the accrual of irreversible joint damage, development of disability, impairment in quality of life, and increased socio-economic costs [2,3,4]. Previous studies reported that up to 47% of PsA patients had radiological damage at a median interval of 2 years from disease onset [5], and even a 6-month delay to the first rheumatologic visit contributes to joint erosion accrual with worse long-term physical function [6].

Since there are no validated biomarkers for PsA, its diagnosis is based primarily on the overall clinical evaluation, which includes the recording of a history, physical examination, and radiographic features [7]. This, together with its wide clinical heterogeneity and the delayed referrals to the rheumatologist, makes the PsA diagnosis highly challenging [8]. 

A particularly critical step for the accurate diagnosis of PsA is the differentiation from other PsA-mimicking arthropathies [9] and nonspecific arthralgia [10]. In PsO patients, having musculoskeletal (MSK) symptoms does not automatically mean having PsA. Indeed, other arthropathies may occur in PsO patients, like in the general population, including degenerative, immuno-mediated, microcrystalline, and central pain sensitization diseases, that may share several clinical features with PsA [11]. Furthermore, there is growing awareness of the presence of a proportion of PsO patients suffering from nonspecific arthralgias, which are defined as unspecific MSK symptoms not attributable to PsA or other concomitant arthropathies [10]. This is an intriguing clinical entity because it may represent a critical phase in the transition process from PsO to PsA, where early intervention may prevent progression to overt PsA [12]. An accurate differential diagnosis of PsA is crucial in PsO patients with MSK symptoms. On the one hand, it prevents misdiagnosis and overtreatment that would expose patients to the worthless risk of adverse effects; on the other hand, it may lead to identifying PsO patients at higher risk of PsA development deserving in-depth monitoring [13].

A close collaboration between dermatologists, who are the first ones to assess and follow up on PsO patients, and rheumatologists, who are responsible for the PsA diagnosis and its differentiation from other mimicking conditions, is strongly recommended [14]. This is why combined clinics are becoming increasingly widespread worldwide [15,16,17]. However, clear evidence of the benefits of a systematic collaboration between the two specialists in managing PsO patients is still scarce, especially regarding the differential diagnosis. Greater knowledge of the prevalence, characteristics, and associated factors of undiagnosed PsA; other PsA-mimicking arthropathies; and unspecific MSK symptoms in PsO patients would provide evidence of the benefits of such collaboration and useful information for its enhancement.

This study aimed to assess the outcomes of a cross-sectional rheumatologic assessment of a large monocentric cohort of PsO patients in terms of the recognition of undiagnosed PsA and its differentiation from other arthropathies and nonspecific arthralgias.

## 2. Materials and Methods

### 2.1. Patients and Study Design

This is the cross-sectional phase of the DIAPASON (Early Diagnosis of PsA in a monocentric cohort of PsO patients) project, an ongoing study developed to prospectively identify the prevalence, predictors, and outcomes of PsA in the context of systematic collaboration between dermatologists and rheumatologists. Overall, 1057 consecutive adult (age ≥18 years) PsO patients, who were followed at the Dermatology Unit of the Azienda Ospedaliero-Universitaria (AOU) of Cagliari from 1 January 2021 to 30 June 2022, underwent a visit by the SpA clinic team (3 expert rheumatology consultants and 3 residents) from the Rheumatology Unit of the same institution. 

According to good clinical practice, as in a real-world setting, the rheumatologic assessment was based on (a) an evaluation of the present and past medical history, mainly searching for musculoskeletal (MSK) symptoms and potentially associated manifestations; (b) a physical examination based on a systematic assessment of swollen and tender joints (respectively, 66 and 68 peripheral joints), active dactylitis, enthesitis, tender points (as listed in the 1990 classification criteria for fibromyalgia) (14), and other signs potentially related to concomitant arthropathies (e.g., Heberden or Bouchard nodules, rheumatoid nodules, tophi, etc.) or another rheumatologic disease potentially causing MSK symptoms; and (c) laboratory (e.g., rheumatoid factor, anti-citrullinated peptide antibody, acute phase reactants, uric acid, HLA-B27) and imaging investigations (X-ray, ultrasounds, or MRI) were performed when clinically appropriate. In particular, according to the European Alliance of Associations for Rheumatology (EULAR) recommendations for the use of imaging in the diagnosis of spondylarthritis in clinical practice, ultrasound was primarily performed on patients with suspected enthesitis, and an MRI was performed to assess the potential axial involvement [18].

This study was approved by the Local Ethical Committee (PROT. NP/2020/4440) of the Azienda Ospedaliero Universitaria of Cagliari. Written informed consent was obtained from all participants.

### 2.2. Outcome Definitions and Data Collection

According to the objective of the study, the following outcomes with their respective definitions were evaluated:

- Previous diagnosis of PsA: Patients with a pre-existing diagnosis of PsA. The previous diagnoses were retrospectively re-evaluated and then confirmed if they were formally made by a rheumatologist and fulfilled the classification criteria for psoriatic arthritis (CASPAR), which were developed and validated for clinical research but are frequently used in clinical practice to guide the clinician in the diagnosis process [19]. In particular, the following clinical criteria were retrospectively evaluated: occurrence of inflammatory articular manifestations, current PsO, personal or family history of PsO, dactylitis, juxta-articular new bone formation in hands or feet X-ray, negative rheumatoid factor, and psoriatic nail dystrophy [19].
- Suspected PsA: Patients without a pre-existing diagnosis of PsA but with suspected PsA based on the rheumatologist’s judgment (according to the presence of manifestations considered as potentially attributable to PsA) and/or an Early Psoriatic Arthritis Screening Questionnaire (EARP) score ≥ 3 [20]. The EARP is a simple self-administered questionnaire consisting of 10 questions that investigate signs or symptoms that are potentially attributable to PsA (presence = 1, absent = 0). A total score ≥ 3 was validated to identify suspected PsA cases [20]. Following the in-depth rheumatologic assessment, the suspected PsA cases were then classified as follows:
  o New PsA diagnosis: a new diagnosis of PsA made according to the CASPAR criteria after the rheumatologic assessment.
  o PsA-alternative disease: Any other diagnosis explaining the recorded MSK symptoms. When available, the other rheumatologic diseases (e.g., fibromyalgia, connective tissue diseases, rheumatoid arthritis, knee osteoarthritis, and gout) were diagnosed according to the currently used and validated diagnostic/classification criteria [21,22,23,24,25,26,27,28,29].
  o Nonspecific arthralgia: arthralgia not explained by PsA [21] or other concomitant diseases.

If a patient received a new diagnosis of both PsA and another arthropathy, they were classified only in the “new PsA diagnosis” group, and the concomitant MSK disease was separately recorded as an overlap. In all recruited patients, the following data were recorded: demographics, date of PsO onset and diagnosis, PsO skin pattern, max body surface area (BSA) involvement during the whole PsO course, familiarity with PsO and PsA, history of uveitis or inflammatory bowel diseases (IBDs), ongoing treatment, and comorbidities. The EARP questionnaire was administered to every patient to identify and classify the self-perceived MSK symptoms. 

In the PsA patients with a new diagnosis of PsA, the following data were also recorded: swollen and tender joint count, PsA pattern, erythrocyte sedimentation rate (ESR), C-reactive protein (CRP), patient global assessment of disease activity (PtGA) and pain on a 10 cm visual analog scale, possible treatment modification following the PsA diagnosis, and the disease activity PsA composite index (DAPSA).

### 2.3. Statistical Analysis

Categorical variables are reported as absolute numbers and frequencies (%). Normally and non-normally distributed continuous variables are reported as mean ± standard deviation (SD) and/or median and interquartile range (IQR), respectively. Univariate logistic regression analysis was performed to identify demographic and clinical variables associated with the new diagnosis of PsA in patients with suspected PsA. The effect size for such an association is expressed as an odds ratio (OR) with a 95% confidence interval (CI). Statistical significance was set at a *p*-value < 0.05.

## 3. Results

### 3.1. Study Population

In total, 1057 patients with PsO were recruited for the present study. There were 585 (55.3%) males, and the mean (SD) age at enrolment was 55.3 (14.7) years, with a mean PsO disease duration of 20.1 (14.8) years (Table 1).

The most prevalent patterns of skin involvement were plaque (84.9%) and palm-plantar PsO (12.5%). Nail involvement was recorded in 504 (47.7%) patients. Details of the patient’s demographic and clinical characteristics are reported in Table 1.

### 3.2. Suspected PsA

**Figure 1:**
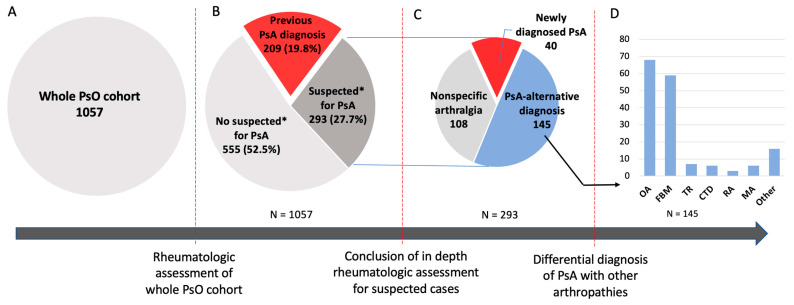
Results of the subsequent phases of the cross-sectional rheumatologic assessment of the whole psoriatic arthritis cohort. (**A**) Before the rheumatologic assessment. (**B**) After the rheumatologic assessment. (**C**) After the conclusion of the in depth rheumatologic assessment of the suspected cases of PsA. (**D**) PsA-alternative diagnoses. PsO, skin and nail psoriasis. PsA, psoriatic arthritis. EARP, early psoriatic arthritis screening questionnaire. OA, osteoarthritis. FBM, fibromyalgia. TR, trauma. CTD, connective tissue disease. RA, rheumatoid arthritis. MA, microcrystalline arthropathy. * Suspicion of PsA was based on rheumatologist’s judgment and or EARP ≥ 3.

In the whole PsO cohort (Figure 1A), 209 (19.8%) patients already had a PsA diagnosis (Figure 1B). Of the remaining 848 patients, 293 (34.6%) were classified as suspected PsA (Figure 1C). In 129 patients, the suspicion was based both on EARP ≥ 3 and the rheumatologist’s judgment; in 164, it was based on the EARP score but not the rheumatologist’s judgment. Only one case was classified as suspected by the rheumatologist despite EARP < 3 (Figure 1B). 

#### 3.2.1. New PsA Diagnosis

A new diagnosis of PsA was made in 40 (13.7%) of the 293 suspected cases (Figure 1C), with an increase in the total disease prevalence from 19.7% to 23.6% in the whole cohort (Figure 1C).

The mean duration of MSK symptoms from their onset to the diagnosis of PsA was 44.6 (44.5) months, with 29 (72.5%) patients having a symptom duration ≥ 1 year. 

The mean EARP score at the rheumatology assessment was 6.2 (1.8). Most of the patients (55.0%) had moderate disease activity (DAPSA score >14 and ≤28). Twenty patients (50.0%) showed active peripheral arthritis, with an oligoarticular pattern in most cases (85.0%). Active dactylitis and enthesitis were recorded in 14 (35.0%) and 4 (10.0%) patients, respectively. Thirteen patients complained of possible inflammatory low back pain, but only four had an axial involvement confirmed via imaging. Details of the demographic and clinical features of the newly diagnosed patients are reported in Table 2. Searching for potential overlaps between PsA and other arthropathies, it was found that out of the patients with a new PsA diagnosis, 14 (35.0%) and 9 (22.5%) patients were also affected by fibromyalgia and osteoarthritis, respectively. Overlap with other arthropathies was not recorded in these patients.

Following the diagnosis of PsA, 34 (85.0%) patients underwent a treatment modification consisting of the introduction or dosage increase in cs-DMARDs in 21 (55.2%) patients and b-DMARDs in 13 (32.5%) cases. In the remaining seven patients whose treatment was not modified by the rheumatologist, it was because the dermatologist already made a recent change with a potential effect also in the MSK component.

#### 3.2.2. PsA-Alternative Diagnosis

Another diagnosis potentially explaining the MSK symptoms was recorded in 142 (48.3%) of the 293 patients with suspected PsA (Figure 1C). In particular, the most frequent PsA-alternative diagnoses were osteoarthritis, which was recorded in 68 patients, fibromyalgia in 59, post-traumatic alterations in 7, microcrystalline arthropathy in 6, connective tissue disease in 6, and rheumatoid arthritis in 3 (Figure 1D). Twenty-four patients had more than one condition that potentially explained their symptoms. Details on the demographic, clinical, and therapeutic characteristics of this sub-group of patients are reported in Table 2.

Overall, the patients with a PsA-alternative diagnosis had a mean (SD) EARP score of 4.9 (1.67). Fifty-one patients (35.1%) needed more than one visit to reach their final diagnosis. All patients received specific recommendations regarding follow-up and pharmacological and non-pharmacological interventions to manage their newly diagnosed condition.

#### 3.2.3. Nonspecific Arthralgia

Out of the 293 patients with suspected PsA, 108 (36.9%) were classified as having nonspecific arthralgia after excluding the presence of PsA and other arthropathies potentially explaining their MSK symptoms (Figure 1C). 

Overall, the patients with unspecific arthralgia had a mean (SD) EARP score of 4.4 (1.6). All patients were informed regarding the importance of surveillance and follow-up for the possible evolution to PsA. Details on the demographic, clinical, and therapeutic characteristics of this sub-group of patients are reported in Table 2.

### 3.3. Factors Differentiating PsA from Nonconfirmed PsA Suspected Cases

**Figure 2:**
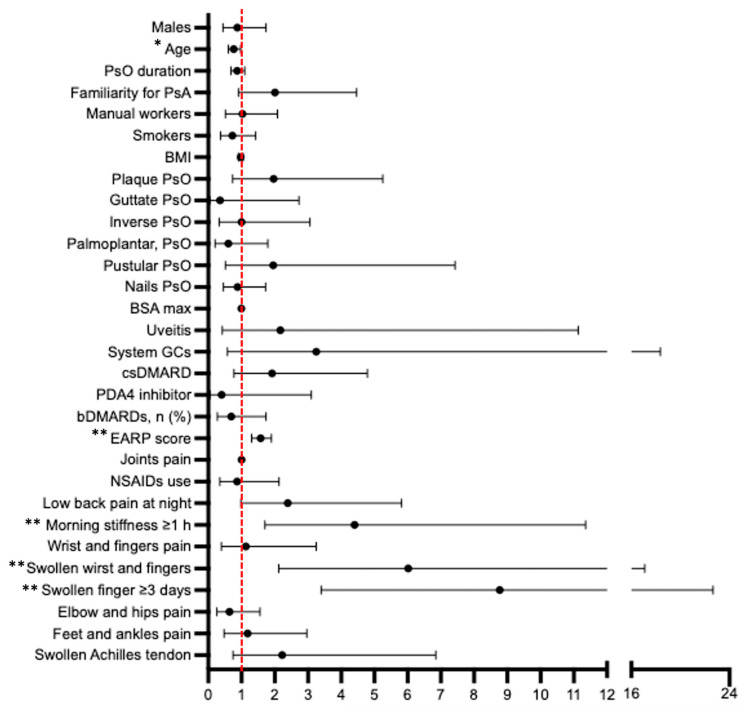
Forest plot representing the odds ratio (OR) of a new PsA diagnosis in PsO patients classified as suspected for joint involvement based on EARP ≥ 3 or rheumatologist’s judgment. PsA, psoriatic arthritis. PsO, psoriasis. BMI, body mass index. BSA, body surface area. GCs, glucocorticoids. EARP, Early Psoriatic Arthritis Screening Questionnaire. cs and b-DMARDs, conventional synthetic and biologic disease-modifying anti-rheumatic drugs, respectively. * *p* < 0.05, ** *p* < 0.001.

Among patients with suspected PsA, the clinical factors significantly associated with a confirmed new diagnosis of PsA were the EARP score (OR 1.574 for a one-unit increase, 95% CI 1.304 to 1.901, *p* < 0.001), swelling in the wrist and fingers (OR 6.021, 95% CI 2.122 to 17.082, *p* < 0.001), and a swollen finger for at least three days (OR 8.775, 95% CI 3.398 to 22.66, *p* < 0.001), as self-reported by the patients in questions 6 and 7 of the EARP questionnaire (Figure 2). 

Conversely, age was negatively associated with a new PsA diagnosis (OR 0.768 for a 10-year increase, 95% CI 0.605 to 0.977). Similar results emerged when comparing the sub-groups of newly diagnosed PsA with the sub-group of patients with PsA-alternative diagnosis and that of patients with nonspecific arthralgia (Supplementary Materials).

## 4. Discussion

Through the implementation of a dermato-rheumatologic multidisciplinary unit (parallel model) [30], this study provides meaningful data on the outcomes of a systematic rheumatologic assessment of PsO patients in terms of the identification of undiagnosed PsA cases and their differentiation from other PsA-mimicking diseases and unspecific arthralgia.

The cross-sectional rheumatology assessment of more than one thousand PsO patients led to 40 new diagnoses of PsA, accounting for 16% of the total cases of PsA, with an increase in the final disease prevalence from 20% to 24%. These data are in accordance with a recent meta-analysis that showed that pooling data from several PsO cohorts estimated 15% of undiagnosed PsA cases and suggested the under-recognition of the joint disease as a significant determinant of its variable epidemiological data [31]. Furthermore, this study demonstrated that three-quarters of the newly diagnosed patients had MSK symptoms longer than one year with a clinical pattern mainly characterized by moderate disease activity, oligoarticular synovitis, and/or dactylitis and/or enthesitis. These results suggest that more attention should be paid to the early recognition of mild and moderate forms of PsA, which deserve adequate treatment like the more severe forms [14,17]. In our analysis, the latter assumption is supported by the demonstration that about 85% of the new PsA diagnoses resulted in an escalation of the ongoing treatment, targeting more comprehensive management of all disease domains.

Regarding the PsO patients with MSK symptoms explained by another diagnosis, these accounted for about half of the suspected PsA cases. In our PsO cohort, the most frequently recorded PsA-alternative diagnoses were osteoarthritis (OA) and fibromyalgia (FBM). However, like in previous reports [32], the association of PsO with other immuno-mediated diseases, including rheumatoid arthritis and connective tissue diseases, was also recorded (most of them were already diagnosed at the time of our study visit). Based on the epidemiology of such conditions, these data were not surprising. Indeed, OA and FBM were expected to be the most frequent arthropathies in our PsO cohort, as well as in the general population [33,34]. On the other hand, the high prevalence of skin PsO in the general population (up to 3%) [35] suggests the potential association with other diseases, such as RA and CTD. Noteworthily, although the high prevalence of these diseases was expected, their differentiation from PsA remains challenging and requires a dermatologist’s and rheumatologist’s expertise. This is especially true since these diseases do not exclude PsA. Indeed, they may coexist, as demonstrated by the overlap with OA and FBM in 22.5% and 35.0% of the new PsA diagnoses in our cohort. Particularly challenging may be the differentiation of PsA from FBM, mainly because of its increased prevalence in psoriatic patients and the problematic discrimination of pain in the entheseal sites. In this regard, according to the emerging evidence on the use of ultrasound (US) in differentiating PsA enthesitis from FBM tenderness [36], the classification of entheseal pain was the most frequent indication for US in our cohort. 

Finally, regarding a PsA-alternative diagnosis in PsO, collaboration between a dermatologist and rheumatologist is critical, not only in the differentiation from PsA but also in patient management. Indeed, these conditions may also require adequate patient information and pharmacological and/or non-pharmacological interventions [37,38]. Furthermore, these patients will need accurate monitoring over time since having an alternative diagnosis does not exclude that they may also develop PsA. The role of the dermatologist will be particularly important in detecting possible modification of the clinical picture and “re-refer” the patient to the rheumatologist.

In the differential diagnosis process, after identifying the undiagnosed PsA cases and the PsA-alternative diagnoses, a remarkable group of 108 patients with nonspecific arthralgias (37% of the suspected PsA cases) was recognized. To our knowledge, this is one of the first studies providing data on the size of this intriguing clinical entity that in a recent Delphi consensus study focused on terminology for preclinical phases of PsA was defined as “psoriasis with MSK symptoms not explained by another diagnosis” [12,39]. This condition is highly interesting in the study of the transition process from the skin to the joint disease since it includes the so-called “prodromal” or “preclinical” PsA phase [31,32]. Savage et al. found that 55% of patients newly diagnosed with PsA had nonspecific MSK symptoms that preceded, by about two years, the onset of the inflammatory symptoms suggestive of PsA [40]. On the other hand, only a variable proportion of these patients will develop PsA, as demonstrated in another study, where arthralgia in women (HR 2.59), heel pain (HR 4.18), high fatigue score (HR 2.36), and high stiffness score (HR 2.03) were reported to predict the subsequent development of PsA in PsO patients [10]. Overall, these data highlight the unmet need for biomarkers that are helpful in predicting and/or monitoring the transition process toward PsA. In this context, recent studies showed that tenosynovitis and enthesitis demonstrated using US underlie a significant proportion of nonspecific arthralgia and are associated with a higher risk of PsA development [41]. The longitudinal phase of the DIAPASON study will provide a further contribution to understanding this sub-group of patients and the respective risk for the transition toward PsA.

Finally, as a further demonstration of the challenging differential diagnosis in PsO patients, we found that in the screening process, the only factors with a significant utility in discriminating PsA from other diseases and nonspecific arthralgias were young age (mainly due to the expected older age recorded in the patients with osteoarthritis, which was the most prevalent PsA-alternative diagnosis) and higher EARP score with a history of morning stiffness, swollen joints, or dactylitis. These data underlie the complementary role of the two specialists. The dermatologist is the first to assess these patients and is responsible for the challenging screening process, where the mere administration of a self-administrated questionnaire may be inadequate because of its relatively low specificity. In our analysis, an EARP ≥ 3 screening had a 100% sensitivity but a 66% specificity. It is difficult to state what should be the acceptable performance of a screening tool since it may depend on the applied model of the multidisciplinary approach and the available resources in terms of physicians, spaces, and time. An ideal model may be the parallel one, where all PsO patients also undergo a rheumatologic assessment at each visit. However, this is not always applicable everywhere. Thus, using a screening questionnaire with an accurate interpretation process performed by an expert dermatologist may prevent excessive and inappropriate referral to the rheumatologist, making most of the collaboration models more sustainable. Moreover, the dermatologist has a critical role in informing and sensitizing PsO patients about possible MSK involvement. The rheumatologist is responsible for the definitive differential diagnosis and the management of PsA, as well as the other possibly diagnosed arthropathies.

This study had some limitations. First, the analysis of a monocentric cohort from a tertiary dermatologic center may prevent the generalization of the results because of the risk of selecting patients with more severe PsO and the broader use of systemic drugs, including b-DMARDs. Second, the retrospective nature of this study prevented a precise distinction between early and established forms of the newly diagnosed PsA cases. Although we tried to mitigate this limitation by distinguishing patients with reported symptom duration < 1 and ≥1 year, the prospective extension of this study is required to provide more specific data about the characteristics of newly onset PsA and undiagnosed established forms. Third, laboratory and imaging investigations, such as ultrasonography, were not prescribed according to a predefined protocol but on a case-by-case basis according to the rheumatologist’s clinical judgment and the current international recommendations [18]. Although this approach mirrors the current clinical practice [18], it prevented a homogeneous availability of some data and then a specific sub-analysis on the effectiveness of these investigations in the differential diagnosis of PsA, as well as in the classification of nonspecific arthralgias. A predetermined laboratory and imaging protocol, including ultrasound examination, will be applied to better characterize and follow up these patients in the prospective phase of the DIAPASON project.

## 5. Conclusions

This study demonstrated a high prevalence of musculoskeletal symptoms suspected for PsA in PsO patients with, however, only a small proportion due to undiagnosed PsA. Overall, this study showed how the clinical heterogenicity of PsA, the high prevalence of other arthropathies, the frequent occurrence of nonspecific arthralgias potentially representing a prodromal phase of PsA, and the relatively poor performance of the screening tools make the differential diagnosis of PsA highly challenging. Furthermore, our results demonstrated how an accurate classification of musculoskeletal symptoms in PsO patients is not a mere academic exercise but a critical phase in the management of these patients since it leads to appropriate treatment, both in patients with newly diagnosed PsA and in those with other arthropathies equally deserving of adequate care. 

Thus, close collaboration between dermatologists and rheumatologists plays a crucial role not only in diagnosing and managing PsA but also in the distinction and management of other musculoskeletal disorders. Follow-up data from these patients will provide further evidence regarding the benefits of the early recognition of the transition from PsO to PsA, especially in patients with nonspecific arthralgia, and an improvement in other long-term outcomes.

## Figures and Tables

**Table 1:** Characteristics of the whole PsO cohort (*n* = 1057).

Demographics	
Males, *n* (%)	585 (55.3)
Age, mean (SD), yrs	55.3 (14.7)
Age at the PsO onset, mean (SD), yrs	35.4 (18.2)
PsO disease duration, mean (SD), yrs	20.1 (14.8)
Familiarity for PsA, *n* (%)	138 (13.1)
Manual workers, *n* (%)	466 (48.0)
Smokers, *n* (%)	670 (63.9)
BMI, mean (SD) kg/m^2^	26.5 (5.0)
**PsO skin pattern**	
Plaque, *n* (%)	897 (84.9%)
Guttate, *n* (%)	53 (5.0%)
Inverse, *n* (%)	89 (8.4%)
Palmoplantar, *n* (%)	132 (12.5%)
Pustular, *n* (%)	32 (3.0%)
Erythroderma, *n* (%)	22 (2.1%)
Nails PsO, *n* (%)	504 (47.7%)
BSA max, mean (SD)	35.4 (18.3)
**SpA-related comorbidities**	
Uveitis, *n* (%)	16 (1.5%)
IBD, *n* (%)	11 (1.0%)
**Ongoing treatment**	
Topic, *n* (%)	753 (71.2%)
Phototherapy, *n* (%)	10 (0.9%)
Systemic glucocorticoids, *n* (%)	27 (2.6%)
cs-DMARDs, *n* (%)	176 (16.7%)
Apremilast, *n* (%)	58 (5.5%)
b-DMARDs, *n* (%)	380 (36.0%)
**Total EARP score, mean (SD)**	2.5 (2.7)
Individual EARP items *	
Joints pains, *n* (%)	342 (58.8)
NSAIDs used twice last 3 months, *n* (%)	104 (17.9)
Low back pain at night, *n* (%)	83 (14.3)
Morning stiffness ≥ 1 h, *n* (%)	100 (17.2)
Wrist and finger pain, *n* (%)	219 (37.6)
Swollen joints, *n* (%)	127 (21.9%)
Swollen wrist and fingers ≥ 3 days, *n* (%)	69 (11.9)
Elbow and hip pain, *n* (%)	123 (21.1)
Feet and ankle pain, *n* (%)	163 (28.0)
Swollen Achilles tendon, *n* (%)	40 (6.9)
**Previous PsA diagnosis** **, *n* (%)**	209 (34.6%)

PsO, skin and nail psoriasis. PsA, psoriatic arthritis. IBD, inflammatory bowel disease. cs-DMARDs, conventional synthetic disease-modifying anti-rheumatic disease. b-DMARDs, biologic DMARDs. EARP, Early Psoriatic Arthritis Screening Questionnaire. * Total score available for all patients; details on individual items for about half the cohort.

**Table 2:** Characteristics of suspected PsA patients grouped according to the final rheumatologist’s classification.

	New PsA Diagnosis (*n* = 40)	Other Diagnosis (*n* = 145)	Unspecific Arthralgia (*n* = 108)
**Demographics**			
Males, *n* (%)	15 (37.5)	53 (36.6)	50 (46.3)
Age, mean (SD), yrs	51.9 (11.6)	58.1 (14.1)	55.4 (13.6)
Age at PsO onset, mean (SD), yrs	34.8 (19.0)	38.1 (18.7)	35.2 (17.9)
PsO duration, mean (SD), yrs	17.1 (13.6)	20.2 (15.5)	20.3 (15.5)
Familiarity for PsA, *n* (%)	10 (25.0)	21 (14.5)	15 (13.9)
Manual workers, *n* (%)	21 (56.7)	78 (53.8)	53 (49.1)
Smokers, *n* (%)	23 (57.5)	89 (61.4)	36 (33.3)
BMI, mean (SD) kg/m^2^	26.1 (4.0)	26.9 (4.9)	26.3 (5.3)
**PsO skin pattern**			
Plaque, *n* (%)	35 (87.5)	111 (76.6)	85 (78.7)
Guttate, *n* (%)	1 (2.5)	14 (9.7)	3 (2.8)
Inverse, *n* (%)	4 (10.0)	16 (11.0)	9 (8.3)
Palmoplantar, *n* (%)	4 (10.0)	22 (15.2)	17 (15.7)
Pustular, *n* (%)	4 (10.0)	4 (2.8)	6 (5.6)
Erythroderma, *n* (%)	0	3 (2.1)	3 (2.8)
Nails PsO, *n* (%)	19 (47.5)	64 (44.1)	63 (58.3)
BSA max, mean (SD)	16.2 (14.2)	18.6 (20.0)	18.9 (21.7)
**SpA-related comorbidities**			
Uveitis, *n* (%)	2 (5.0%)	5 (3.4)	1 (0.9)
IBD, *n* (%)	0	2 (1.4)	1 (0.9)
**Ongoing treatment**			
Topic, *n* (%)	35 (87.5)	117 (80.7)	84 (77.8)
Phototherapy, *n* (%)	0	2 (1.4)	2 (1.9)
Systemic glucocorticoids, *n* (%)	2 (5.0)	1 (0.7)	3 (2.8)
cs-DMARD, *n* (%)	7 (17.5)	12 (8.3)	13 (12.0)
PDA4 inhibitor, *n* (%)	1 (2.5)	11 (7.6)	4 (3.7)
b-DMARDs, *n* (%)	6 (15.0)	28 (19.3)	23 (21)
**Total EARP, mean (SD) score**	6.2 (1.7)	4.9 (1.7)	4.4 (1.6)
Individual EARP items *			
Joints pain, *n* (%)	23 (100)	84 (100)	66 (95.7)
NSAIDs used twice last 3 months, *n* (%)	8 (34.8)	39 (4.4)	20 (29.0)
Low back pain at night, *n* (%)	11 (47.8)	27 (32.1)	16 (23.2)
Morning stiffness ≥ 1 h, *n* (%)	16 (69.6)	32 (38.1)	21 (30.4)
Wrist and finger pain, *n* (%)	18 (78.3)	67 (79.8)	51 (73.9)
Swollen joints, *n* (%)	18 (78.3)	38 (45.2)	20 (29.0)
Swollen wrist/fingers ≥ 3 days, *n* (%)	13 (56.5)	13 (15.5)	7 (10.1)
Elbow and hip pain, *n* (%)	9 (39.1)	41 (48.8)	37 (53.6)
Feet and ankle pain, *n* (%)	15 (65.2)	55 (66.5)	40 (58.0)
Swollen Achilles tendon, *n* (%)	5 (21.7)	9 (10.7)	8 (11.6)
**Therapy modification after PsA diagnosis** **, *n* (%)**	34 (85.9)	-	-
Introduction cs-DMARD, *n* (%)	21 (55.2)	-	-
Introduction b-DMARD, *n* (%)	13 (32.5)	-	-
Injection joint therapy, *n* (%)	0	-	-

PsO, skin and nail psoriasis. PsA, psoriatic arthritis. IBD, inflammatory bowel disease. cs-DMARDs, conventional synthetic disease-modifying anti-rheumatic disease. b-DMARDs biologic DMARDs. EARP, Early Psoriatic Arthritis Screening Questionnaire. * Total score available for all patients; details on individual items for about half cohort.

## Data Availability

The study dataset is not publicly available, but it is available from the corresponding author upon reasonable request.

## Supplementary Materials

The following supporting information can be downloaded from https://www.mdpi.com/article/10.3390/jcm12186090/s1: Table S1. Comparison of characteristics of suspected PsA patients grouped according to the final rheumatologist’s classification.

## References

[1] FitzGerald O., Ogdie A., Chandran V., Coates L.C., Kavanaugh A., Tillett W., Leung Y.Y., de Wit M., Scher J.U., Mease P.J. (2021). Psoriatic arthritis. Nat. Rev. Dis. Primer.

[2] Siannis F. (2006). Clinical and radiological damage in psoriatic arthritis. Ann. Rheum. Dis..

[3] Kavanaugh A., Helliwell P., Ritchlin C.T. (2016). Psoriatic Arthritis and Burden of Disease: Patient Perspectives from the Population-Based Multinational Assessment of Psoriasis and Psoriatic Arthritis (MAPP) Survey. Rheumatol. Ther..

[4] Wervers K., Luime J.J., Tchetverikov I., Gerards A.H., Kok M.R., Appels C.W.Y., van der Graaff W.L., van Groenendael J.H.L.M., Korswagen L.-A., Dieren J.J.V. (2018). Influence of Disease Manifestations on Health-related Quality of Life in Early Psoriatic Arthritis. J. Rheumatol..

[5] Kane D., Stafford L., Bresnihan B., FitzGerald O. (2003). A prospective, clinical and radiological study of early psoriatic arthritis: An early synovitis clinic experience. Rheumatol. Oxf. Engl..

[6] Haroon M., Gallagher P., FitzGerald O. (2015). Diagnostic delay of more than 6 months contributes to poor radiographic and functional outcome in psoriatic arthritis. Ann. Rheum. Dis..

[7] Mease P., Goffe B.S. (2005). Diagnosis and treatment of psoriatic arthritis. J. Am. Acad. Dermatol..

[8] Gratacós J., Behrens F., Coates L.C., Lubrano E., Thaçi D., Bundy C., de la Torre-Aboki J., Luelmo J., Voorneveld H., Richette P. (2021). A 12-point recommendation framework to support advancement of the multidisciplinary care of psoriatic arthritis: A call to action. Jt. Bone Spine.

[9] Rida M.A., Chandran V. (2020). Challenges in the clinical diagnosis of psoriatic arthritis. Clin. Immunol..

[10] Eder L., Polachek A., Rosen C.F., Chandran V., Cook R., Gladman D.D. (2017). The Development of Psoriatic Arthritis in Patients With Psoriasis Is Preceded by a Period of Nonspecific Musculoskeletal Symptoms: A Prospective Cohort Study. Arthritis Rheumatol..

[11] Ritchlin C.T., Colbert R.A., Gladman D.D. (2017). Psoriatic Arthritis. N. Engl. J. Med..

[12] Scher J.U., Ogdie A., Merola J.F., Ritchlin C. (2019). Preventing psoriatic arthritis: Focusing on patients with psoriasis at increased risk of transition. Nat. Rev. Rheumatol..

[13] Landewé R.B.M. (2018). Overdiagnosis and overtreatment in rheumatology: A little caution is in order. Ann. Rheum. Dis..

[14] Gossec L., Baraliakos X., Kerschbaumer A., de Wit M., McInnes I., Dougados M., Primdahl J., McGonagle D.G., Aletaha D., Balanescu A. (2020). EULAR recommendations for the management of psoriatic arthritis with pharmacological therapies: 2019 update. Ann. Rheum. Dis..

[15] Luchetti M.M., Benfaremo D., Campanati A., Molinelli E., Ciferri M., Cataldi S., Capeci W., Di Carlo M., Offidani A.M., Salaffi F. (2018). Clinical outcomes and feasibility of the multidisciplinary management of patients with psoriatic arthritis: Two-year clinical experience of a dermo-rheumatologic clinic. Clin. Rheumatol..

[16] Theodorakopoulou E., Dalamaga M., Katsimbri P., Boumpas D.T., Papadavid E. (2020). How does the joint dermatology–rheumatology clinic benefit both patients and dermatologists?. Dermatol. Ther..

[17] Coates L.C., Soriano E.R., Corp N., Bertheussen H., Callis Duffin K., Campanholo C.B., Chau J., Eder L., Fernández-Ávila D.G., FitzGerald O. (2022). Group for Research and Assessment of Psoriasis and Psoriatic Arthritis (GRAPPA): Updated treatment recommendations for psoriatic arthritis 2021. Nat. Rev. Rheumatol..

[18] Mandl P., Navarro-Compán V., Terslev L., Aegerter P., van der Heijde D., D’Agostino M.A., Baraliakos X., Pedersen S.J., Jurik A.G., Naredo E. (2015). EULAR recommendations for the use of imaging in the diagnosis and management of spondyloarthritis in clinical practice. Ann. Rheum. Dis..

[19] Taylor W., Gladman D., Helliwell P., Marchesoni A., Mease P., Mielants H. (2006). CASPAR Study Group Classification criteria for psoriatic arthritis: Development of new criteria from a large international study. Arthritis Rheum..

[20] Tinazzi I., Adami S., Zanolin E.M., Caimmi C., Confente S., Girolomoni G., Gisondi P., Biasi D., McGonagle D. (2012). The early psoriatic arthritis screening questionnaire: A simple and fast method for the identification of arthritis in patients with psoriasis. Rheumatology.

[21] Neogi T., Jansen T.L.T.A., Dalbeth N., Fransen J., Schumacher H.R., Berendsen D., Brown M., Choi H., Edwards N.L., Janssens H.J.E.M. (2015). 2015 Gout classification criteria: An American College of Rheumatology/European League Against Rheumatism collaborative initiative. Ann. Rheum. Dis..

[22] Wolfe F., Clauw D.J., Fitzcharles M.-A., Goldenberg D.L., Häuser W., Katz R.L., Mease P.J., Russell A.S., Russell I.J., Walitt B. (2016). 2016 Revisions to the 2010/2011 fibromyalgia diagnostic criteria. Semin. Arthritis Rheum..

[23] Dasgupta B., Cimmino M.A., Maradit-Kremers H., Schmidt W.A., Schirmer M., Salvarani C., Bachta A., Dejaco C., Duftner C., Jensen H.S. (2012). 2012 provisional classification criteria for polymyalgia rheumatica: A European League Against Rheumatism/American College of Rheumatology collaborative initiative. Ann. Rheum. Dis..

[24] Aletaha D., Neogi T., Silman A.J., Funovits J., Felson D.T., Bingham C.O., Birnbaum N.S., Burmester G.R., Bykerk V.P., Cohen M.D. (2010). 2010 Rheumatoid arthritis classification criteria: An American College of Rheumatology/European League Against Rheumatism collaborative initiative. Arthritis Rheum..

[25] Aringer M., Costenbader K., Daikh D., Brinks R., Mosca M., Ramsey-Goldman R., Smolen J.S., Wofsy D., Boumpas D.T., Kamen D.L. (2019). 2019 European League Against Rheumatism/American College of Rheumatology classification criteria for systemic lupus erythematosus. Ann. Rheum. Dis..

[26] Altman R., Alarcón G., Appelrouth D., Bloch D., Borenstein D., Brandt K., Brown C., Cooke T.D., Daniel W., Feldman D. (1991). The American College of Rheumatology criteria for the classification and reporting of osteoarthritis of the hip. Arthritis Rheum..

[27] Altman R., Asch E., Bloch D., Bole G., Borenstein D., Brandt K., Christy W., Cooke T.D., Greenwald R., Hochberg M. (1986). Development of criteria for the classification and reporting of osteoarthritis. Classification of osteoarthritis of the knee. Diagnostic and Therapeutic Criteria Committee of the American Rheumatism Association. Arthritis Rheum..

[28] Altman R., Alarcon G., Appelrouth D., Bloch D., Borenstein D., Brandt K., Brown C., Cooke T.D., Daniel W., Gray R. (1990). The American College of Rheumatology criteria for the classification and reporting of osteoarthritis of the hand. Arthritis Rheum..

[29] Zhang W., Doherty M., Peat G., Bierma-Zeinstra M.A., Arden N.K., Bresnihan B., Herrero-Beaumont G., Kirschner S., Leeb B.F., Lohmander L.S. (2010). EULAR evidence-based recommendations for the diagnosis of knee osteoarthritis. Ann. Rheum. Dis..

[30] Gudu T., Jadon D.R. (2020). Multidisciplinary working in the management of axial and peripheral spondyloarthritis. Ther. Adv. Musculoskelet. Dis..

[31] Villani A.P., Rouzaud M., Sevrain M., Barnetche T., Paul C., Richard M.-A., Beylot-Barry M., Misery L., Joly P., Le Maitre M. (2015). Prevalence of undiagnosed psoriatic arthritis among psoriasis patients: Systematic review and meta-analysis. J. Am. Acad. Dermatol..

[32] Wu J.J., Nguyen T.U., Poon K.-Y.T., Herrinton L.J. (2012). The association of psoriasis with autoimmune diseases. J. Am. Acad. Dermatol..

[33] Cooper G.S., Stroehla B.C. (2003). The epidemiology of autoimmune diseases. Autoimmun. Rev..

[34] Martel-Pelletier J., Barr A.J., Cicuttini F.M., Conaghan P.G., Cooper C., Goldring M.B., Goldring S.R., Jones G., Teichtahl A.J., Pelletier J.-P. (2016). Osteoarthritis. Nat. Rev. Dis. Primer.

[35] Ogdie A., Weiss P. (2015). The Epidemiology Psoriatic Arthritis. Rheum. Dis. Clin. North Am..

[36] Marchesoni A., Macchioni P., Gasparini S., Perricone C., Perrotta F.M., Grembiale R.D., Silvagni E., Ramonda R., Costa L., Zabotti A. (2021). Use of Ultrasonography to Discriminate Psoriatic Arthritis from Fibromyalgia: A Post-Hoc Analysis of the ULISSE Study. J. Clin. Med..

[37] Kolasinski S.L., Neogi T., Hochberg M.C., Oatis C., Guyatt G., Block J., Callahan L., Copenhaver C., Dodge C., Felson D. (2020). 2019 American College of Rheumatology/Arthritis Foundation Guideline for the Management of Osteoarthritis of the Hand, Hip, and Knee. Arthritis Care Res..

[38] Macfarlane G.J., Kronisch C., Dean L.E., Atzeni F., Häuser W., Fluß E., Choy E., Kosek E., Amris K., Branco J. (2017). EULAR revised recommendations for the management of fibromyalgia. Ann. Rheum. Dis..

[39] Perez-Chada L.M., Haberman R.H., Chandran V., Rosen C.F., Ritchlin C., Eder L., Mease P., Reddy S., Ogdie A., Merola J.F. (2021). Consensus terminology for preclinical phases of psoriatic arthritis for use in research studies: Results from a Delphi consensus study. Nat. Rev. Rheumatol..

[40] Savage L., Tinazzi I., Zabotti A., Laws P.M., Wittmann M., McGonagle D. (2020). Defining Pre-Clinical Psoriatic Arthritis in an Integrated Dermato-Rheumatology Environment. J. Clin. Med..

[41] Zabotti A., McGonagle D.G., Giovannini I., Errichetti E., Zuliani F., Zanetti A., Tinazzi I., Lucia O.D., Batticciotto A., Idolazzi L. (2019). Transition phase towards psoriatic arthritis: Clinical and ultrasonographic characterisation of psoriatic arthralgia. RMD Open.

