# Supplementary material for: The Challenging Differentiation of Psoriatic Arthritis from Other Arthropathies and Nonspecific Arthralgias in Patients with Psoriasis: Results of a Cross-Sectional Rheumatologic Assessment of a Large Dermatologic Cohort
Source: J Clin Med. 2023 Sep 21;12(18):6090. doi: 10.3390/jcm12186090 (PMC10532027; doi:10.3390/jcm12186090)
Supplement: Supplementary file 1 [file jcm-12-06090-s001.zip › jcm-2570020-supplementary.pdf]

Supplementary Material

Table S1. Comparison of characteristics of suspected PsA patients grouped according to the final rheumatologist's classification

	New PsA diagnosis (A, n= 40)	Other diagnosis (B, n= 145)	Unspecific arthralgia (C, n= 108)	A vs A+B	A vs B	A vs C
**Demographics**						
Males, n (%)	15 (37.5)	53 (36.6)	50 (46.3)	n.s.	n.s.	n.s.
Age, mean (SD), yrs	51.9 (11.6)	58.1 (14.1)	55.4 (13.6)	**0.029**	**0.001**	n.s.
Age at PsO onset, mean (SD) yrs	34.8 (19.0)	38.1 (18.7)	35.2 (17.9)	n.s.	n.s.	n.s.
PsO duration, mean SD) yrs	17.1 (13.6)	20.2 (15.5)	20.3 (15.5)	n.s.	n.s.	n.s.
Familiarity for PsA, n (%)	10 (25.0)	21 (14.5)	15 (13.9)	n.s.	n.s.	n.s.
Manual workers, n (%)	21 (56.7)	78 (53.8)	53 (49.1)	n.s.	n.s.	n.s.
Smokers, n (%)	23 (57.5)	89 (61.4)	36 (33.3)			
BMI	26.1 (4.0)	26.9 (4.9)	26.3 (5.3)	n.s.	n.s.	n.s.
**PsO skin pattern**						
Plaque, n (%)	35 (87.5)	111 (76.6)	85 (78.7)	n.s.	n.s.	n.s.
Guttate, n (%)	1 (2.5)	14 (9.7)	3 (2.8)	n.s.	n.s.	n.s.
Inverse, n (%)	4 (10.0)	16 (11.0)	9 (8.3)	n.s.	n.s.	n.s.
Palmoplantar, n (%)	4 (10.0)	22 (15.2)	17 (15.7)	n.s.	n.s.	n.s.
Pustular, n (%)	4 (10.0)	4 (2.8)	6 (5.6)	n.s.	n.s.	n.s.
Erythroderma, n (%)	0	3 (2.1)	3 (2.8)	n.s.	n.s.	n.s.
Nails PsO, n (%)	19 (47.5)	64 (44.1)	63 (58.3)	n.s.	n.s.	n.s.
BSA max, mean (SD)	16.2 (14.2)	18.6 (20.0)	18.9 (21.7)			
**SpA-related Comorbidities**						
Uveitis, n (%)	2 (5.0%)	5 (3.4)	1 (0.9)	n.s.	n.s.	n.s.
IBD, n (%)	0	2 (1.4)	1 (0.9)	n.s.	n.s.	n.s.
**Ongoing treatment**						
Topic, n (%)	35 (87.5)	117 (80.7)	84 (77.8)	n.s.	n.s.	n.s.
Phototherapy, n (%)	0	2 (1.4)	2 (1.9)	n.s.	n.s.	n.s.
System. glucocorticoids, n (%)	2 (5.0)	1 (0.7)	3 (2.8)	n.s.	n.s.	n.s.
csDMARD, n (%)	7 (17.5)	12 (8.3)	13 (12.0)	n.s.	n.s.	n.s.
PDA4 inhibitor, n (%)	1 (2.5)	11 (7.6)	4 (3.7)	n.s.	n.s.	n.s.
bDMARDs, n (%)	6 (15.0)	28 (19.3)	23 (21)	n.s.	n.s.	n.s.
**Total EARP score**	6.2 (1.7)	4.9 (1.7)	4.4 (1.6)	**<0.001**	**<0.001**	**<0.001**
**Individual EARP items***						
Joints pain	23 (100)	84 (100)	66 (95.7)	n.s.	n.s.	n.s.
NSAIDs use twice last 3 months	8 (34.8)	39 (4.4)	20 (29.0)	n.s.	n.s.	n.s.
Low back pain at night	11 (47.8)	27 (32.1)	16 (23.2)	0.051	0.005	0.001
Morning stiffness ≥1 hour	16 (69.6)	32 (38.1)	21 (30.4)	0.001	n.s.	n.s.
Wrist and fingers pain	18 (78.3)	67 (79.8)	51 (73.9)	n.s	n.s.	n.s.
Swollen joints	18 (78.3)	38 (45.2)	20 (29.0)	**<0.001**	**0.005**	**<0.001**
Swollen wrist / fingers ≥3 days	13 (56.5)	13 (15.5)	7 (10.1)	**<0.001**	**<0.001**	**<0.001**
Elbow and hips pain	9 (39.1)	41 (48.8)	37 (53.6)	n.s.	n.s.	n.s.
Feet and ankles pain	15 (65.2)	55 (66.5)	40 (58.0)	n.s.	n.s.	n.s.
Swollen Achilles tendon	5 (21.7)	9 (10.7)	8 (11.6)	n.s.	n.s.	n.s.
**Treat. mod. after PsA diagn.**	34 (85.9)	-	-	-	-	-
Introduction csDMARD	21 (55.2)	-	-	-	-	-
Introduction bDMARD	13 (32.5)	-	-	-	-	-
Injection joint therapy	0	-	-	-	-	-

PsO, skin and nail psoriasis. PsA, Psoriatic arthritis. IBD, inflammatory bowel disease. csDMARDs, conventional synthetic disease-modifying anti-rheumatic disease. bDMARDs biologic DMARDs.
